# From Waste to Value: Fish Protein Hydrolysates as a Technological and Functional Ingredient in Human Nutrition

**DOI:** 10.3390/foods13193120

**Published:** 2024-09-29

**Authors:** Adrián Honrado, Marta Miguel, Paula Ardila, José Antonio Beltrán, Juan B. Calanche

**Affiliations:** Instituto Agroalimentario de Aragón-IA2, Universidad de Zaragoza-CITA, Miguel Servet 177, 50013 Zaragoza, Spain; adrihonfri@unizar.es (A.H.); ardilap@unizar.es (P.A.); jbeltran@unizar.es (J.A.B.)

**Keywords:** fish protein hydrolysates, bioactive compounds, technological properties, sensory characterization, nutritional characteristics

## Abstract

Fish provides a low-caloric content, polyunsaturated fatty acids, many essential trace elements and is also a rich source of protein, ranging from 10% to 25%. Therefore, the production of FPH (fish protein hydrolysates) is of great interest, as the resulting products exhibit a variety of important bioactive and technological properties, making them potential ingredients for new functional foods and supplements. The aim of this review was to compile and analyze information on enzymatic hydrolysates, with particular emphasis on those derived from fish by-products, as a potential ingredient in human nutrition. Their nutritional characteristics, food safety aspects, bioactive properties, technological attributes, key influencing factors, and applications in food products were evaluated. The findings revealed that these properties are influenced by several factors, such as the raw material, enzymes used, degree of hydrolysis, and the molecular weight of the peptides, which need to be considered as a whole. In conclusion, the gathered information suggests that it is possible to obtain high-value products through enzymatic hydrolysis, even when using fish by-products. However, although numerous studies focused on FPH derived from fish muscle, research on by-products remains limited. Further investigation is needed to determine whether the behavior of FPH from by-products differs from that of muscle-derived FPH.

## 1. Introduction

Fish ranks as one of the most consumed foods worldwide, owing to its high nutritional value [1]. It is recommended to consume between three to four servings of fish per week, alternating between white and oily fish [2]. In 2020, the estimated global aquatic production was 178 million tons, with 51% from capture fisheries (90 million tons) and the remaining 49% from aquaculture (88 million tons) [3]. Global fish consumption was 20.2 kg per capita in 2020, and a 15% increase is expected, reaching 21.4 kg per capita by 2030 [3]. The European Union reached a per capita consumption of 23.5 kg/hab in 2020, 2.6% less than the previous year [4].

Fish provides low caloric intake [5] and is a rich source of proteins, ranging between 10% and 25% [6]. It contains essential amino acids such as tryptophan, lysine, methionine, and threonine, enhancing digestibility [7]. In 2019, aquatic food intake represented approximately 17% of global animal protein and 7% of total protein. Fish is also notable for its lipid composition, being rich in PUFAs (polyunsaturated fatty acids) such as EPA (eicosapentaenoic acid), DHA (docosahexaenoic acid) and DPA (docosapentaenoic acid), which offer cardiovascular and other health benefits [1,4]. Additionally, fishery products are an exceptional source of vitamins (A, B, D, and E) and minerals (calcium, phosphorus, iodine, zinc, iron, and selenium) [4]. However, the chemical composition of fish may vary depending on muscle type, sex, age, season, habitat, and diet [8].

Regarding seafood end use, in 2020, only 89% was destined for direct human consumption, with over 20 million tons used for non-food purposes [3]. Presently, due to increase per capita consumption [6], substantial amounts of by-products are generated: viscera, heads, skins, and bones, representing 60% to 70% of the total fish weight [6,9]. Most fish processing waste is considered low-value products [10] and often ends up being discharged into the sea or landfills [11], incurring economic and environmental costs [12]. Some fish by-products unsuitable for direct human consumption are used for fishmeal and fish oil production and in feed or fertilizer manufacturing [3]. However, it is known that all these products contain bioactive compounds and essential nutrients capable of benefiting potential consumers’ health [12].

Hence, there is a need to appropriately use both discarded whole fish and generated by-products [13]. It is known that, with proper handling of these by-products, high-value products can be obtained [10] for use in the production of nutraceutical and functional foods [11,14], thus improving waste management; minimizing environmental impact; enhancing the profitability of the fishing industry [9]; and contributing to sustainable development goals: zero hunger, responsible production and consumption, and life below water [3]. Nevertheless, there are limitations related to seasonality, maintaining the optimal freshness of by-products, establishing the necessary infrastructure and required technology in certain regions, or negative consumer perception, which need to be addressed beforehand.

Currently, fishmeal and fish oil are the most used products derived from fish by-product valorization [15] and have been extensively studied and used in animal feed [16]. They are obtained from whole fish or fractions such as heads, skins, or viscera [17]. Fish oil is a liquid-textured product and a rich source of polyunsaturated fatty acids (EPA and DHA) [3,16]. However, the unsaturated nature of the oil makes it unstable and susceptible to oxidative deterioration, negatively affecting the future product’s acceptability when consumed by humans and requiring techniques such as encapsulation or the use of antioxidant substances to avoid nutritional loses and undesirable flavors [18,19].

Considering that fish by-products contain 10% to 20% of the fish’s total protein, another alternative lies in hydrolysis methods, resulting on a mixture of non-hydrolyzed protein, free amino acids, and peptides that may have bioactive properties [20]. Thus, the production of FPHs (fish protein hydrolysates) becomes interesting, as they exhibit various important bioactive and technological properties [16], making them potential ingredients for new functional foods [10].

FPHs are compounds made from the protein fraction of fish muscle, although, in recent times, the use of by products such as viscera, muscle remnants, or skin for this purpose has intensified [10,21]. They are typically found in the form of cream-colored powder, with a characteristic fish odor [16]. This process involves the breakdown of proteins into amino acids and peptides [22], being the bioactive ones usually formed by sequences of 2 to 20 amino acids [23,24].

FPHs can be obtained through processes such as acid or alkaline hydrolysis, bacterial fermentation, or enzymatic hydrolysis [21]. Chemical hydrolysis is cheap and fast [25], but the use of acids and alkalis affects the nutritional quality of the resulting peptides [26], lower hydrolysis yields are obtained, deterioration of generated amino acids is produced, low repeatability is also observed [25], and it can generate a considerable number of salts and unwanted side reactions, such as tryptophan degradation and racemization [27].

Conversely, bacterial fermentation promotes the development of LAB (lactic acid bacteria) that produce acidic and antimicrobial substances, inhibiting competing bacteria. However, this method complicates lipid phase removal [28]. Thus, enzymatic hydrolysis is preferred, as it allows better control over process conditions and does not generate chemical residues [11]. Nevertheless, numerous factors influence the characteristics and quality of hydrolysates or peptides produced. These factors include protein source, amino acid sequence and composition, molecular weight, enzyme type, and chemicals used, as well as temperature, pH, or hydrolysis time [10,29].

Considering the existing research, enzymatic hydrolysis is considered the most effective method for producing FPHs from fish protein with bioactive and functional properties, suitable for human food production [30]. During the hydrolysis process, both the substrate type and enzyme concentration, as well as the hydrolytic conditions, need to be considered [31,32]. Generally, protease enzymes are used to cleave large peptides into smaller fractions [32]. Based on the mechanism of action and catalytic site, enzymes are classified as exoproteases and endoproteases [33]. Exoproteases or exopeptidases decompose the peptide bond at the amino-terminal (N-terminal) or carboxyl-terminal (C-terminal) group, while endoproteases or endopeptidases break the peptide bond in the middle of the protein molecule [12]. Thus, the DH (degree of hydrolysis) quantifies the enzymatic degradation of fish protein [34] and represents the percentage of free amino groups cleaved [13], being a vital indicator for assessing hydrolysis efficiency [35].

Hence, enzymatic hydrolysis emerges as a promising method for producing FPHs and bioactive peptides from fish protein. The nutritional and functional quality of these products makes them potentially valuable in the pharmaceutical and food industry. FPHs are a versatile ingredient in both food technology and human health due to its ability to fortify any food in which an increase in protein content is pursued [36]. They have certain technological properties, such as emulsifying, stabilizing, water retention, and gelling [37]. On the other hand, its sensory characteristics make it also suitable as a flavor enhancer [38]. In addition, its bioactive peptides have various functions in food preservation and disease prevention [27].

Therefore, this study aimed to conduct a review on the utilization of fish protein—especially that from by-products but not exclusively—to produce FPHs with a potential use in the food industry and human consumption. Specific objectives were outlined, including sensory and nutritional characterization of hydrolysates, identification of conditions to ensure food safety (especially of those ones coming from fish by-products), and analysis of bioactive and technological properties, as well as exploration of potential applications in the food industry.

## 2. Materials and Methods

**Figure 1:**
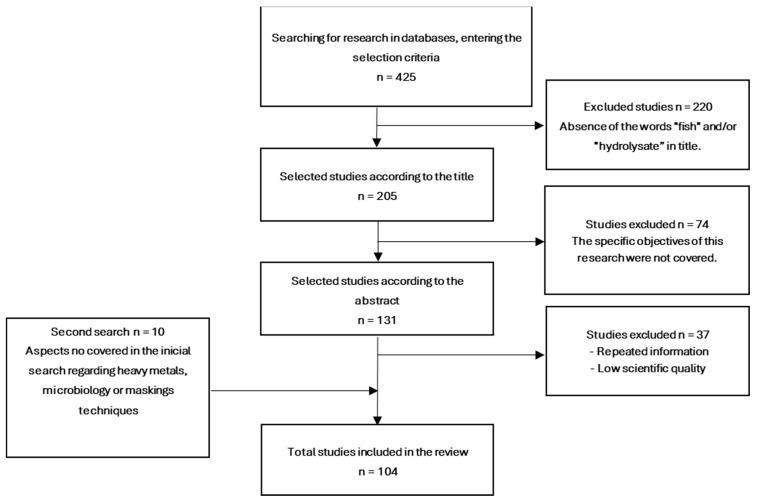
Systematic methodology followed in this research.

The present study was conducted following a systematic review methodology (Figure 1). The selected time range comprised the last five years. The introduced keywords were as follows: fish by-products, fish protein hydrolysates, bioactive peptides, food safety, enzyme, nutritional, properties, functional, technological, composition, application, hydrolysis, innovative, contaminant, and novel food. Information was searched mainly in English across different databases: Google Scholar, PubMed, Science Direct, and Springer Link, among others, and other organizations such as the FAO or WHO. A total of 425 articles were found, which were subsequently selected by title. Those in which the words fish or hydrolysate were not present or were simply not related to the study topic were rejected, reducing the number to 205 articles. Subsequently, abstracts were read to discard those in which the field of study was beyond the scope of this review, resulting in 131 articles. Duplicate information was removed, leaving a total of 94 studies selected for this review. However, as the search progressed, it became apparent that particular aspects which were considered important had not been picked up in the initial search. Therefore, a second search was carried out using words such as heavy metals, microbiology, or encapsulation. This increased the final number of articles to 104. After selecting the documents containing the required information for the review, the results were structured based on the six specific objectives outlined earlier. Once the results were presented and discussed, conclusions were drawn in response to each aim proposed in the previous section.

## 3. Findings to Date

**Figure 2:**
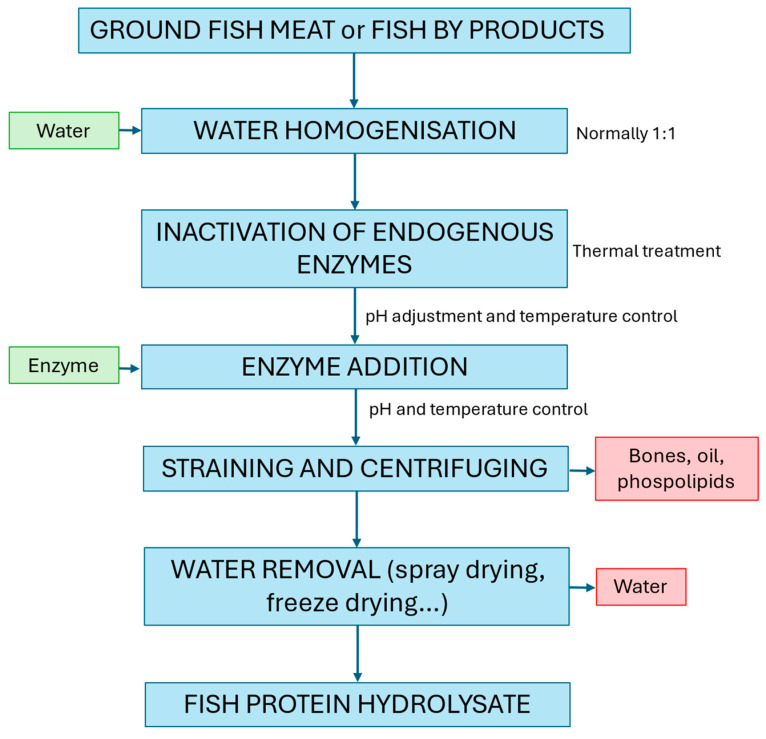
Basic fish enzyme hydrolysates production process [40].

Currently, several processes are employed in the production of FPHs, with fermentation, chemical, and enzymatic hydrolysis being the most important ones. However, enzymatic hydrolysis is a rapid, safe, and easily controllable method to produce FPHs, enhancing their biological and functional properties in comparison to the initial substrates. The commercial proteases used can be derived from microbial sources (e.g., Flavourzyme^®^, Alcalase^®^, plants (e.g., bromelain or ficin), or animals (e.g., trypsin or pepsin). The bioactivity of FPHs depends on several factors, such as the type of protein; pretreatments; enzymatic specificity; and hydrolysis conditions (pH, time, temperature, solid–liquid ratio, and enzyme–substrate ratio). Enzymatic specificity, determined by the amino acid sequence around the cleavage site, and hydrolysis conditions influence the sequences and sizes of the peptides produced. Different conditions and types of enzymes result in various biological activities due to the peptides generated. However, a drawback is the high cost of some enzymes [39]. A basic scheme to produce FPHs throughout enzymatic hydrolysis can be seen in Figure 2.

### 3.1. Sensory and Color Characterization of FPHs

The sensory aspect of FPHs is of great importance, mainly when intended for use in food enrichment and fortification. Despite this significance, there is a scarcity of in-depth studies on this aspect and related to the quality of the existing ones.

One of the most recurrent and studied sensory issues is the occurrence of a bitter taste [41]. Bitterness in FPHs is primarily influenced by hydrophobicity, DH, MW (molecular weight), peptide aminoacidic sequences, the type of enzyme used in the hydrolysis process, and the specific fish by-products utilized [42,43]. Hydrophobicity refers to the presence of free hydrophobic amino acids and peptides containing hydrophobic amino acids, with the latter being the main contributor to bitterness [42]. Studies have shown that a higher DH indicates a greater extent of protein degradation, leading to an increased presence of hydrophobic peptides [33,34,44]. This is also related to MW, as higher degrees of hydrolysis produce lower MW peptides. Recent studies have demonstrated that peptides with MW in the range of 0.5–2 kDa tend to exhibit more intense bitterness [45]. However, the type of enzyme used is the most important aspect, as it conditions the aforementioned factors: different enzymes can yield peptides with varying levels of hydrophobicity, DH, MW, and amino acid sequences [41].

To understand the influence of different enzymes on the flavor of tilapia PH (protein hydrolysates), Gan et al. [33] conducted a study resulting in various flavors, depending on the enzyme used. In tilapia skin PH, the authors reported that Neutrase^®^ yielded higher umami and acidic flavor intensity, while papain resulted in the highest bitterness. In tilapia spine PH, papain led to a sourer and bitter taste, while bromelain produced the highest umami flavor. In tilapia head PH, Alcalase^®^ enzyme stood out for providing the highest umami flavor, papain provided the most acidic flavor, and Neutrase imparted the highest bitterness. A somewhat proportional relationship was found between the DH and bitter taste, but this research also made evident the effect of the composition of the raw material used. In general, the effect that different enzymes have on flavor is related to the enzyme’s specificity. While some enzymes act on specific bonds, others have a more non-specific nature, affecting the final composition of peptides and free amino acids. In this sense, longer peptides may have less intense flavors than smaller peptides or free amino acids, which can contribute stronger flavors such as umami (glutamic acid); bitterness (hydrophobic amino acids like leucine or peptides with these amino acids at their ends); or sweetness (glycine, threonine, or alanine). It is also important to consider the conditions under which hydrolysis is performed, as they can influence the enzyme’s effectiveness [45]. In any case, if the production of FPHs is intended for human consumption, high DH should not be applied, as this situation increases the bitterness of the final product [41]. However, this fact can determine their functional and technological properties [6,33].

Bui et al. [8] pointed out that the optimal DH to gain the best sensory quality falls within the range between 5 and 20% (resulting in peptides of 3 to 10 kDa. It has also been observed that peptides with a characteristic basic taste are grouped around a certain MW: peptides with umami flavor had a MW < 1.5 kDa, and the most acidic flavor peptides had a MW between 1 and 1.5 kDa, while the MW of peptides with the highest bitterness were in the range 2–6 kDa [33,46].

Currently, in response to the limitation in the use of FPH due to undesirable tastes and odors, different methods have been proposed to remove bitterness, minimize these results, and enable their performance in the food industry [34]. This has led to the study of exopeptidase enzymes capable of reducing bitterness by removing terminal hydrophobic amino acids [34]. However, it is noteworthy that the use of additional exopeptidases may result in changes in bioactivity and functional properties and the fact that specific sequences that are part of a peptide also provide a bitter taste and are not affected by the use of exopeptidases [42,44]. For that reason, other techniques have been investigated. Among the methods evaluated to eliminate bitterness from FPHs, encapsulation technique stands out. This technology, in addition to masking the bitterness of FPHs, can mask the characteristic fish odor [43]. Kumari et al. [47] found that microencapsulation through lyophilization was effective in improving the overall acceptability of FPHs from the head and viscera of Pink Perch (*Nemipterus japonicus*). The problem is the feasibility of bringing these encapsulation processes to a large scale.

From another point of view, sight could be considered the sense that best allows consumers to quickly assess the quality of a product, with color being one of the key factors in determining their acceptability [32]. In the case of FPHs, color is influenced by the raw material used, processing method, and reaction conditions such as time or type of enzyme used [32,48]. However, sensory color determination is not common, and most measurements are made instrumentally.

As mentioned, the hydrolysis method significantly influences the color of FPHs. Hassan et al. [13] produced different PHs from pangasius (*Pangasianodon hypophthalmus*) viscera. They performed enzymatic hydrolysis (using papain or pepsin) and chemical hydrolysis (acid or alkaline). Significant differences (*p* < 0.05) were observed in the obtained CIELAB values, with darker and more brownish tones observed in the chemical FPHs, possibly due to the formation of unwanted by-products during the chemical process. However, considering the high levels of pigments present in viscera, it is believed that the resulting color in the FPHs was due to these compounds remaining soluble after the centrifugation process [13].

To verify that the type of enzyme used also influenced the color of the FPHs, Alahmad et al. [49] produced bighead carp (*Hypophthalmichthys nobilis*) muscle PHs with ficin, which exhibited a yellowish coloration. Hydrolysis was carried out using the enzyme ficin, and different DH were achieved. In a subsequent study, Alahmad et al. [32] produced carp muscle PHs using Flavourzyme^®^. At similar DH compared to the previous study, higher values were obtained in the a* and b* coordinates, resulting in redder and more yellowish tones (Table 1). This behavior could be related to the enzyme’s specificity and therefore to the peptides generated and their size. Additionally, it should be noted that some enzymes can break down structures containing pigments, such as hemoglobin or myoglobin. If these pigments are released, the hydrolysates could become darker or reddish.

As it could be seen in other research, Karoud et al. [31] checked that hydrolysis conditions influenced the color of the final product, as hydrolysates obtained from hake (*Merluccius merluccius*) heads presented higher L* when obtained at a lower DH (Table 1). They suggested that the resulting darker color could be due to the oxidation of myoglobin and melanin pigment, present in both the raw material and the final product. Protein oxidation could also be a factor to consider [31,50].

To examine the influence of pH, time, and temperature conditions on the enzymatic process, Pavarthy et al. [48] hydrolyzed red muscle proteins of tuna (*Euthynnus affinis*) using papain. The parameters used were pH, temperature, and time of 6.5, 55 °C, and 45 min. The authors obtained tuna protein hydrolysates with a creamy yellowish appearance and L*, a*, and b* values of 90.97 ± 0.05, −0.83 ± 0.01, and 16.14 ± 0.01, respectively. In another study, Unnikrishnan et al. [35] obtained red tuna (*Thunnus albacares*) muscle PHs, also using papain. This time, they increased the temperature by 5 °C and extended the hydrolysis time to 240 min. They obtained FPHs with a creamy white color and remarkably similar values to those obtained by Pavarthy et al. [48]. In general, it has been observed that the conditions applied in hydrolysis significantly influence the final color of the studied FPHs, including the type of enzyme, hydrolysis duration, pH, and process temperature. Instrumental control of the color of PHFs and the variables that affect it is of vital importance, especially when they are intended for the fortification of existing foods, as very noticeable changes in color would lead to rejection by the consumer.

### 3.2. Proximate Composition

The proximate composition of by-products may be considered the factor on which the nutritional quality of the FPHs depends [35]. However, the type of hydrolysis, as well as other processing parameters, determine the nutritional quality [51].

The main component of interest in FPHs are protein, peptides and amino acids [35]. Several studies have specified that FPHs contain between 60 and 90% protein [48], the reasons for these high protein percentages being the solubilization of proteins during hydrolysis, the removal of insoluble solids by centrifugation, and moisture reduction by different techniques such as spray-drying or freeze-drying (Table 2) [31].

The influence of the hydrolysis method can be observed in some studies, such as that of Hassan et al. [13], which compared enzymatic hydrolysis of *Pangasianodon hypophthalmus* viscera with papain or pepsin with chemical hydrolysis in basic or acidic medium. The protein content decreased significantly with chemical methods, possibly due to the degradation and loss of some protein fractions as a result of the pH, time, and treatment temperature used. This led to an increase in fat and ash content. The increase in ash content is also due to the incorporation of an acid or a base in the case of chemical hydrolysis [13].

The amino acid composition of the FPHs has also been analyzed. Generally, glutamine or glutamic acid stands out in the amino acid composition, which is usually found in fish products and has the highest concentration within the amino acid composition of FPHs [53]. Hasani et al. [54] analyzed the amino acid composition present in mackerel (*Rastrelliger kanagurta*) skin and head FPHs using two different enzymes: Alcalase^®^ and Flavourzyme^®^. They obtained a higher content of amino acids in comparison to raw fish, with the highest concentrations being glutamic acid (12.55% and 11.79%, respectively), aspartic acid (7.99% and 6.98%, respectively), and arginine (7.55% and 8.21%, respectively). They also presented hydrophobic amino acids such as glycine, proline, and phenylalanine, among others. Vieria et al. [55] also obtained essential amino acids such as histidine (5.20%), tyrosine (2.90%), or methionine (1.52%).

Sinthusamran et al. [34] produced salmon (*Salmo salar*) frame PHs with two different enzymes: Alcalase^®^ and Flavourzyme^®^. Glutamine had the highest concentration after the bitterness removal process, with values for FPHs with Alcalase^®^ and Flavourzyme^®^ of 14.98 and 15.14 g/100 g, respectively. It should be noted that different enzymes can give rise to different free amino acid profiles. The total content of essential and non-essential amino acids was similar for both FPHs. Several studies have reported a total of essential amino acids around 50% [35,53,54,56].

There are no significant variations when comparing the amino acid pattern between the by-product and the FPH [35]. However, the protein source influences the composition of essential amino acids present in the FPHs [53]. The slight differences that appear in the composition of the FPHs are influenced by the specificity of the enzyme and the conditions of the hydrolytic process [10]. Table 3 shows a comparison between the total amino acid profiles of different FPHs.

Generally, all studies analyzing the proximal composition of FPHs indicate a high nutritional value, most of them containing important amounts of essential amino acids. Therefore, the use of fish protein hydrolysates would be of great interest in human nutrition.

Additionally, although less significant due to their trace presence, the existence of polyunsaturated fatty acids in fish is notable, especially concentrated in certain areas such as the head, gills, and intestines, among others [57]. Their consumption has been associated with a reduced risk of cardiovascular diseases, as they can decrease triglyceride and plasma triglyceride concentrations, platelet aggregation, and blood pressure [58].

### 3.3. Food Safety

One of the factors influencing food safety and quality is the water activity (a_w_) of the food. Karoud et al. [31] obtained hake (*Merluccius merluccius*) head PHs with low a_w_ values, ranging from 0.192 to 0.201. The lowest a_w_ value was achieved with a DH of 7.7% (1 h). In another study, the produced bighead carp (*Hypophthalmichthys nobilis*) PHs had a variable a_w_ between 0.27 and 0.34. On this occasion, the minimal a_w_ was obtained with a DH of 25.48% (3 h) [32]. No correlation between the DH and a_w_ was observed. The resulting a_w_ values in both studies were within the ideal range, as values between 0.50 and 0.60 could lead to a harder and lower quality product [32]. At this a_w_, the available water is limited and tends to bind more strongly to the food components, which can lead to the formation of bonds between molecules, such as proteins, resulting in a more rigid and less flexible structure. Similarly, higher a_w_ values could promote the development of bacteria, fungi, and yeasts [31].

The microbiological quality of the FPHs is also important from a food safety perspective. Gómez and Zapata [59] analyzed both the by-product and their FPHs from red tilapia (*Oreochromis* spp.). They indicated that the viscera showed a high count of mesophilic aerobic microorganisms (3.71 log CFU/g), total coliforms (450 MPN/g), and molds and yeasts (2.30 log CFU/g). However, reductions of 92.88%, 90%, and 95% were achieved in the produced FPHs from these viscera, respectively. This reduction is a result of the temperature and pH conditions typically applied in the manufacturing of FPHs (elevated temperatures during hydrolysis, degreasing, enzyme inactivation, or drying). On the other hand, the histamine content obtained by Roldán et al. [60] in whole anchovy (*Engraulis ringens*) PHs was 14.91 mg/kg, a very low content considering the maximum limit (100 mg/kg) according to Commission Regulation (EC) no. 2073/2005 of 15 November 2005 on Microbiological Criteria for Foodstuffs [61]. However, is necessary to remark that good manufacturing practices are essential to obtain hydrolysates with maximum food safety.

Furthermore, there is currently limited information regarding heavy metal contamination in fish by-products, as only the edible parts have been considered a risk to human health [62]. However, FPHs being subjected to a concentration process during drying could become a risk. In a study by Ramilo-Fernández and Sotelo [63], the quantification of heavy metals present in the muscle of Southwest Atlantic butterfish (*Stromateus brasiliensis*) was carried out, and the results obtained in this species for mercury, lead, and cadmium were 0.038 ± 0.033 mg/kg, 0.006 ± 0.007 mg/kg, and 0.018 ± 0.017 mg/kg, respectively. In a subsequent work, de la Fuente [62] quantified the concentration of heavy metals in different sea bass edible parts and by-products (muscle, head, viscera, skin, and tail fin). The authors obtained the highest concentration of mercury in the muscle (0.106 ± 0.001 μg/g) and the lowest in the viscera (0.014 ± 0.0003 μg/g). Lead was obtained in a lower concentration in the muscle (0.027 ± 0.0002 μg/g), with the highest content reached in the head (0.063 ± 0.010 μg/g). Regarding cadmium, the highest value was determined in the viscera (0.028 ± 0.0003 μg/g) and the lowest in the muscle (0.001 ± 0.00001 μg/g). In addition, the authors determined the arsenic content in these by-products, obtaining concentrations between 0.687 and 1.867 μg/g. Finally, the research by Donnarumma et al. [64] evaluated the content of metals in tuna by-products, considered one of the species with the greatest tendency to accumulate metals because it is at the end of the trophic chain [65]. Values of 5.74 mg/kg were found for arsenic and 0.45 mg/kg for mercury. The maximum arsenic limit is not regulated in fish. In these studies, the concentrations of Hg, Pb, and Cd remained below the maximum limits established for the meat of these fish in Commission Regulation (EU) 2023/915 of 25 April 2023 on Maximum Levels for Certain Contaminants in Food and Repealing Regulation (EC) no. 1881/2006 [66]. It should be noted that several factors are involved in the accumulation of heavy metals in fish. For example, the presence of water contaminated with heavy metals originating from industry, the age and size of the fish, and its position in the food chain, among others.

Research determining the content of heavy metals present in FPHs are less common. Mangano et al. [67] obtained concentrations of arsenic, mercury, lead, and cadmium of 2.70 ± 0.1 mg/kg, 0.04 ± 0.01 mg/kg, 0.02 ± 0.01 mg/kg, and 0.25 ± 0.05 mg/kg, respectively, in anchovy (*E. encrasicholus*) viscera PHs. Similarly, they determined the concentrations of chromium (0.31 ± 0.1 mg/kg), cobalt (0.04 ± 0.01 mg/kg), and nickel (0.55 ± 0.2 mg/kg). In another study, de la Fuente et al. [68] worked with hydrolyzed mackerel (*Scomber scombrus*) by-products. The authors indicated that the most abundant heavy metal was arsenic (0.969–1.379 μg/g) in mackerel PHs, followed by cadmium (0.124–0.150 μg/g), lead (0.048–0.104 μg/g), and mercury (0.044 μg/g). In salmon (*Salmo salar*) by-product PHs, they also obtained higher concentrations of arsenic (1.107–1.421 μg/g), and, in lesser proportions, they quantified lead (0.083–0.093 μg/g), mercury (0.029–0.047 μg/g), and cadmium (0.006–0.015 μg/g). All analyzed heavy metals were below the maximum limits for fish meat established in Regulation (EU) 2023/915, except for Cd in mackerel PHs (0.10 mg/kg). Furthermore, de la Fuente et al. [68] indicated that the presence of other contaminants, such as dioxins and PCBs (polychlorinated biphenyls), in the analyzed FPHs should be low because of fat reduction during hydrolysis.

In conclusion, to ensure the food safety of FPHs, it is crucial to comply with the legal requirements that allow these by-products to be used for human consumption. This includes applying controlled conditions during processing to eliminate pathogens and contaminants without compromising their bioactive and functional properties, the use of fresh and high-quality fish sources, and the maintenance of the cold chain. Keeping strict hygienic conditions at all stages of the process is also key to ensuring the safety of the final product. Similarly, the origin of the fish used can have an important effect on the food safety of FPHs. The publications above show low a_w_ values, allowing for good food product stability and increased shelf life [32]. Additionally, the microbiological analysis of the FPHs reveals that these products are safe for use in food [59]. The previous results indicate that FPHs are not considered a potential risk based on levels of exposure to heavy metals [69].

### 3.4. Bioactive Properties

Biologically active peptides are known to affect human health positively [43]. The production of FPHs has allowed the evaluation of the biological properties that these compounds may possess, with several factors determining bioactivity [6,70].

#### 3.4.1. Antioxidant Activity

The process of lipid oxidation is the primary cause of deterioration in foodstuff, leading to rancidity and decreased shelf life [71]. The most effective method to prevent rancidity involves the use of antioxidants [71]: active chemical substances with the ability to intercept, combat, prevent, or reduce ROS (reactive oxygen species) through various mechanisms [43], with increasing emphasis on antioxidants not originating from chemical synthesis. However, FPHs could also be utilized for this purpose [72], as they are capable of exhibiting antioxidant action through various mechanisms, including the reduction of hydroperoxides, scavenging of free radicals, and chelation of metal ions [71].

Several studies have investigated the difference in free radical scavenging capacity depending on enzymatic hydrolysis conditions such as pH, temperature, and time. Guo et al. [25] observed that increasing the pH from 6.5 to 7 increased the antioxidant activity of catfish (*Pterygoplichthys disjunctivus*) muscle PHs; however, at pH 8, the antioxidant action decreased. This is because these pH values are optimal for enzyme action and favor the release of peptides containing amino acids with antioxidant capacity, such as glutamic acid and other aliphatic amino acids. Moreover, antioxidant peptides generated by enzymatic hydrolysis of proteins vary in their ability to donate electrons or protons, chelate metals, or neutralize free radicals, depending on their chemical structure, which, in turn, is affected by pH. At more alkaline pHs, greater hydrolysis is commonly favored, releasing more active peptides with antioxidant activity. However, too high or too low pH can lead to the degradation of certain amino acids or alter the conformation of proteins, decreasing their antioxidant capacity. It is believed that pH variation influences electrostatic interactions between charged amino acids, breaking the present hydrogen bonds. These pH changes cause amino acid deamination and racemization [73].

Similarly, antioxidant activity increased with the increasing temperature and decreased after 50 °C. Additionally, heat action leads to degradation or aggregation of the antioxidant peptide and exposure of hydrophobic amino acids [35].

Different enzymes significantly influence the MW of peptides and amino acid sequences, which, in turn, depend on the raw material used, thus conditioning their antioxidant activity [34]. It has also been found that process time influences radical scavenging capacity. Generally, increasing the duration of enzymatic hydrolysis increases the DH, reducing the MW of peptides and thus increasing antioxidant activity [54]. It has been demonstrated that peptides with lower MW, especially those below 3000 Da, can more easily donate electrons or hydrogen atoms and react with free radicals to form more stable compounds [37,74]. Furthermore, antioxidant action depends on the type of amino acids and their position within the peptide sequence [23]. In this regard, the presence of hydrophobic and aromatic amino acids (phenylalanine, tryptophan, and tyrosine) increases the antioxidant activity of the resulting hydrolysates [10,35]. Aromatic amino acids are known to donate protons to electron-deficient radicals, maintaining their stability and improving radical removal. Moreover, peptides containing the amino acid tyrosine within their sequence have been shown to have higher antioxidant activity [75]. For example, FPHs produced by Rabiei et al. [52] from Klunzinger’s mullet (*Liza klunzingeri*) muscle contained serine (9.59%), tyrosine (8.43%), cysteine (7.20%), and valine (6.60%) in the PH with the highest antioxidant activity via the DPPH radical scavenging method. Similarly, Saidi et al. [30] observed hydrophobic amino acid residues such as alanine, glycine, isoleucine, tyrosine, tryptophan, and proline in the peptide sequences of tuna black muscle PHs. It is important to take into account the use of debittering techniques that may influence the antioxidant capacity of FPH. Debittering techniques, such as the use of alcohols (2-butanol and isopropanol), affect the antioxidant capacity of protein hydrolysates by influencing the antioxidant activity, depending on the method used. The study by Sinthusamran et al. showed that, after debittering, there was a reduction in the antioxidant activity of salmon frames PHs, especially in terms of ABTS radical scavenging capacity and metal chelating activity, compared to unbittered hydrolysates. The reason could be related to the removal of hydrophobic peptides or amino acids that contribute to both bitterness and antioxidant activity [34].

#### 3.4.2. Antihypertensive Activity

Hypertension is a global risk factor, as it increases the likelihood of developing coronary diseases, strokes, and other cardiovascular-related issues. Typically, hypertension is managed with antihypertensive medications and lifestyle changes in the population [24]. Most of these medications are based on ACE (angiotensin-converting enzyme) inhibition [72].

Recently, several studies have demonstrated the ACE inhibitory activity of PHs derived from fish by-products. Hassan et al. [13] indicated that the antihypertensive activity depended on the MW, amino acid sequence, and the hydrophobicity of peptides present in the FPH. Additionally, differences in ACE inhibition activities of FPHs from different by-products of the same species, such as heads, trims, and viscera of turbot (*Scophthalmus maximus*), have been observed [76], indicating that the amino acid composition and sequence influence the antihypertensive properties.

Again, enzyme specificity is a factor to consider. Qara and Habibi Najafi [77] found that whole ribbon fish (*Lepturacanthus savala*) FPHs generated with Alcalase^®^ and Neutrase^®^ and containing peptides with a MW below 10 kDa exhibited higher inhibition activity than those produced with pepsin and Protamex^®^. Conversely, hydrolysates with pepsin and Protamex^®^ showed no inhibitory activity, as they probably contained high MW peptides (>30 kDa), demonstrating that peptides with lower MW have better access to the ACE active site [78]. Yathisha et al. [78] produced FPHs from a mixture of by-products of Skipjack tuna (*Katsuwonus pelamis*) at different times (1 h, 2 h, 3 h, 4 h, and 5 h) with Alcalase^®^ enzyme and found that, as the hydrolysis time increased and, therefore, the DH, the ACE inhibition activity increased (36.50%, 57.92%, 62.79%, 69.30%, and 75.74%, respectively). These authors also suggested that low MW peptides are more effective at inhibiting ACEs.

Furthermore, the presence of hydrophobic and aromatic amino acids also determines the ACE inhibitory activity [79]. Naghdi, Rezaei et al. [80] obtained the highest ACE inhibition activity in skipjack tuna (*Katsuwonus pelamis*) head PHs, which contained a high content of phenylalanine and tyrosine, unlike PHs from skin and bones. According to Qara and Habibi Najafi [77], this enzyme is strongly influenced by the C-terminal tripeptide sequence of the substrate or inhibitor. ACE appears to prefer substrates or competitive inhibitors that mainly have hydrophobic amino acid residues at the three C-terminal positions.

Therefore, it has been demonstrated that a range of factors such as the raw material, type of enzyme, time, and DH significantly influence the antihypertensive activity of FPH. Production conditions must be optimized, as these factors determine the MW, composition, and sequence of amino acids, which, in turn, are responsible for a higher or lower level of ACE inhibitory activity. In general, different studies have shown that enzymatic PHs from fish by-products would be an effective alternative for preventing and controlling arterial hypertension.

#### 3.4.3. Antimicrobial Activity

FPHs contain antimicrobial peptides, which consist of amino acid chains smaller than 10 kDa and 50 amino acids, with half being hydrophobic. These peptides are typically amphipathic, rich in cysteine residues, and possess a positive charge in their active form, allowing them to exert antimicrobial effects against bacterial cells, yeast, fungi or viruses [81]. It is known that these peptides can induce bacterial cell death due to their ability to form pores or block membrane ion gradients [21]. The formation of pores disrupts the integrity and permeability of the membrane, leading to impairment of cell respiration, interference with the electrochemical gradient, and influx of water and ions, ultimately resulting in cell swelling and lysis. Although scarce, several studies have demonstrated that peptides obtained from the hydrolysis of fish by-products possess antibacterial activity against many Gram-negative and Gram-positive strains [7]. However, this antimicrobial activity depends on the amino acid sequence, MW, and structural characteristics such as hydrophobicity, among others [23].

Hydrolysis time has been proven to be a factor determining the antimicrobial efficacy of FPH, as it conditions the DH [54,70]. Specifically, several studies have determined greater effectiveness in FPHs with a predominant presence of peptides with a MW of less than 3 kDa [23]. Additionally, the type of enzyme used, as well as the raw material or part of the fish used (head, bones, viscera, or skin), also seems to affect the antimicrobial activity [77]. Table 4 shows a comparison of the antimicrobial capacity of different FPHs obtained using different by-products and conditions.

It can be concluded that, although the species used or the enzyme may have an effect on the antimicrobial capacity, longer hydrolysis times and therefore higher DH and lower MW of the peptides have a crucial impact on antimicrobial effectiveness.

#### 3.4.4. Anticancer Activity

Cancer is considered the leading cause of death worldwide, responsible for nearly 10 million deaths in 2020 [82]. It is known that the consumption of foods rich in natural antioxidants can prevent the onset of cancer by inhibiting free radicals and ROS [52]. Several studies have evaluated the antiproliferative and anticancer activities of various PHs [43]. Thus, it has been found that FPHs contain bioactive peptides with anticancer potential [12].

Rabiei et al. [52] produced enzymatic PHs of *Liza klunzingeri* muscle with cytotoxic activities on the 4T1 breast cancer cell line. The authors noted that the sample with the highest cytotoxic activity was the one obtained at a shorter hydrolysis time. In another study, Hasani et al. [54] also reported the relationship between hydrolysis time and the level of anticancer activity. They indicated that the Alcalase^®^ enzyme produced the highest anticancer activity (78.71%) at 30 min in PHs from mackerel shaho (*Rastrelliger kanagurta*) by-products. On the other hand, Shahosseini et al. [74] investigated the HT-29 colon cancer cell line. The authors found that using different enzymes at different DHs (21.34% with Flavourzyme^®^, 28.10% with Alcalase^®^, and 36.45% with Alcalase^®^-Flavourzyme^®^), the PHs from mullet fish (*Liza abu*) muscle exhibited different anticancer activities. However, no clear correlation has been determined between the DH and the antiproliferative activity [83]. Nevertheless, it has been shown that the cytotoxic effects exhibited by PHs depend on the protein source, type and concentration of the enzyme, and hydrolysis process conditions [52]. Yaghoubzadeh et al. [84] investigated the anticancer activity in fractionated rainbow trout (*Oncorhynchus mykiss*) skin PH. Hydrolysis was conducted using Alcalase^®^ and Flavourzyme^®^ enzymes, obtaining PHs with MW between 3.3 and 30 kDa. They investigated the viability (%) of the HCT-116 colon cancer cell line with different concentrations of these FPHs. The authors noted that the half-maximal inhibitory concentration (IC_50_) was obtained with the PH with a MW less than 3 kDa, demonstrating that molecular size also influenced the cytotoxic effect. This information was corroborated by Shahosseini et al. [74], who hydrolyzed *Liza abu* muscle and obtained higher anticancer activity (82.75%) in those FPHs under 3 kDa. In addition to MW, the amino acid composition also influences anticancer activity. This is because the bioactivity level can be increased depending on the high content of hydrophobic peptides at the N-terminal end [83]. However, the number of studies investigating the potential anticancer activity of FPHs is limited, even more in fish by-products PHs, and more information on the mechanism of action of these compounds against cancer cells is needed.

#### 3.4.5. Antidiabetic Activity

Diabetes is a metabolic disorder responsible for the deaths of 2 million people in 2019 [85]. This disease is characterized by high levels of blood glucose as a result of insulin resistance or insufficient secretion of it [86]. Assorted studies have evaluated the antidiabetic activity of proteins and peptides derived from fish [87].

It has been demonstrated that inhibiting the enzymes responsible for blood glucose regulation, such as AAM (α-amylase), AG (α-glucosidase), and DPP-IV (dipeptidyl peptidase), is the most effective strategy for controlling type 2 diabetes [86]. Some studies have evaluated the inhibition of DPP-IV in FPH. It is known that DPP-IV inhibitors prevent the degradation of incretin hormones, GLP-1 (glucagon-like peptide-1), and GIP (glucose-dependent insulinotropic polypeptide), resulting in increased insulin secretion [87]. It has been reported that free amino acids such as glutamine stimulate incretin secretion; however, it is unknown whether this increase is due to greater breakdown of peptides into free amino acids [88].

In one study, Harnedy-Rothwell et al. [89] evaluated the ability of industrially produced blue whiting (*Micromesistius poutassou*) minced meat hydrolysates to inhibit DPP-IV under different hydrolysis conditions. The authors noted that all generated PHs were capable of inhibiting the DPP-IV enzyme. Furthermore, they observed a decrease in DPP-IV inhibition activity of most FPHs after simulated gastrointestinal digestion. This indicates that the peptides responsible for inhibiting DPP-IV were degraded and lost activity during digestion. However, all analyzed samples still exhibited inhibitory activity (IC_50_ = 2.39–2.80 mg/mL).

On the other hand, Henriques et al. [90] evaluated the inhibitory activity of AAM and AG in PHs prepared from different samples (heads, skins, spines, and whole fish) of various fishes (blue whiting, hake, redfish, pout, sand eel, and mackerel). The authors noted differences in the inhibition capacity of different FPHs, as in the case of AAM, where the IC_50_ values ranged from 5.70 to 84.37 mg/mL, while, for AG, the IC_50_ values ranged from 21.8 to 300 mg/mL. These differences were due to the MW and amino acid composition. The specific mechanism by which fish proteins and FPH or bioactive peptides exhibit antidiabetic activity remains unclear [87]. There are few recent studies on the antidiabetic activity of FPH.

### 3.5. Technological Properties

The application of hydrolysis treatment on proteins influences their functional properties. Hydrolysis determines the MW, hydrophobicity, and polar groups of the FPH [91]. These characteristics significantly affect the technological properties of the hydrolysates [43], as the reduction in peptide size and the increase in carboxyl groups of amino acids simplify the protein structure and thus improve the functional quality and bioavailability [28]. Among the functional properties of FPHs, solubility, emulsifying and foaming properties, water-holding capacity, and oil absorption capacity stand out [7].

#### 3.5.1. Solubility

Solubility serves as a significant indicator of the functionality of FPHs [13]. It has been demonstrated that pH, DH, amino acid composition, and MW determine the solubility values of the generated FPHs [47]. Specifically, an increase in solubility has been observed with the increase in pH. This is attributed to the negative net charge of amino acids that rises as the pH value increases, leading to enhanced ionic and hydrogen bonding with water [47,92]. It is noteworthy that other studies, such as the one of Alahmad et al. in bighead carp (*Hypophthalmichthys nobilis*) minced raw fish, have found lower solubility at a pH close to the Ip (isoelectric point) of protein molecules. These decreases occur because, at the molecule’s Ip (pH 4.5 to 5.5), there is a weak interaction between the protein and water. Consequently, moving the pH value away from the Ip enhances this interaction, thus increasing solubility [32].

The DH of FPHs is also related to their solubility, as protein hydrolysis produces smaller peptide fractions [93]. Consequently, the reduction in MW exposes more polar and ionizable groups, thereby increasing solubility [28,32]. On the other hand, Sinthusamran et al. indicated that “debittered” PHs were less soluble, suggesting that hydrophobic amino acids may be the primary contributors to this property. Table 5 presents a series of studies that support the findings [34].

#### 3.5.2. FPHs Foaming Capacity

Foam formation is attributed to the unfolding of peptides and the orientation of the hydrophobic group near the gas phase and the hydrophilic group near the liquid phase [47]. It has been observed that hydrophobicity, pH, temperature, and DH influence the foaming properties of FPHs [56]. Numerous studies have determined the FC (foaming capacity) and FS (foam stability) of generated FPHs.

Greyling et al. [56] indicated that the FC of PHs from monkfish (*Lophius vomerinus*) heads increased from 9 to 20% as the hydrolysis temperature increased. Additionally, Naghdi et al. indicated that the FC of PHs from carp heads (*Hypophthalmichthys nobilis*) increased with the increasing pH value, being higher at pH 9 [70]. In another study, the tuna head-derived FPHs exhibited the highest FC and FS values at pH 7 [80]. Also, Halim and Mhd Sarbon [95] indicated significant differences (*p* < 0.05) in the stability and foaming capacity of produced Asian swamp eel (*Monopterus* sp.) flesh PHs. The authors obtained the highest FC (92.50%) and FS (61.67%) at pH 7 and the lowest foaming capacities at pH 4. However, they observed a decrease as the pH increased from 7 to 10. This is because molecules at pH 7 have good ordering, where the hydrophilic group is in the liquid phase and the hydrophobic group faces non-polar components, thus resulting in a more stable foam.

Karoud et al. evaluated the FC and FS of hake head PHs at different concentrations (0.5%, 1%, and 2%). The authors found that increasing the degree of hydrolysis decreased the foaming properties. With a low MW, FPHs are unable to maintain the well-ordered interfacial orientation of the molecule [93]. For example, in the study by Mohanty et al. using PHs produced from Rohu (*Labeo rohita*) viscera, it was demonstrated that the FC followed a linear behavior that was inversely proportional to the degree of hydrolysis, with a decrease of approximately 10% when the degree of hydrolysis increased by 10% [96]. Thus, it is possible to form a more stable foam when FPHs contain larger peptides [80].

#### 3.5.3. Emulsification Capacity

FPHs are surfactant materials that promote oil-in-water emulsion since they are soluble in water and present hydrophilic and hydrophobic groups [13]. To verify the emulsifying properties of these products, various studies have determined the EAI (emulsifying activity index) and the ESI (emulsion stability index) of different FPHs. Kumari et al. [47] analyzed the EAI and ESI of FPHs from Pink Perch (*Nemipterus japonicus*) by-products at different concentrations (0.5%, 1%, and 2%). The authors indicated that the EAI decreased, and the ESI increased with the increasing FPH concentration. It was observed that, at lower concentrations, proteins had greater ease in diffusing and adsorbing at the oil–water interface, which improved emulsion formation. However, at higher concentrations, the high protein density in the solution could lead to the formation of aggregates or saturation at the interface, which hinders the stability of the emulsion and reduces the efficiency of its formation [47]. Additionally, pH also influences the emulsifying properties of FPHs due to the change of the charges in the peptides [47]. Possibly, emulsifying properties increase at alkaline pH because peptides unfold due to negative charges. Thus, the resulting repulsion promotes the orientation of hydrophilic and hydrophobic peptides at the oil–water interface [70]. Naghdi et al. [80] obtained the highest indices at pH 9 in by-products (skin, bones, and head) of Skipjack tuna (*Katsuwonus pelamis*). Conversely, Kumari et al. [47] found that the increasing pH also increased the EAI when testing Pink Perch (*Nemipterus Japonicus*) by-products FPHs. However, the ESI only increased up to pH 6 and abruptly decreased at the alkaline pH. The lowest indices obtained by Naghdi et al. [70] bighead (*Hypophthalmichthys nobilis*) viscera PHs were at pH 5 (Ip), possibly due to association with hydrophobic amino acids. The DH also seems to have some effect: increasing the DH decreases the EAI and ESI. Smaller peptides are considered less effective in stabilizing emulsions [13]. Alahmad et al. [49] investigated the EAI and ESI of bighead carp (*Hypophthalmichthys nobilis*) FPHs produced with Ficin at different concentrations (0.1%, 0.5%, and 1%) and different degrees of hydrolysis (13.36%, 17.09%, and 20.15%), also corroborating the aforementioned points.

#### 3.5.4. Water- and Oil-Holding Capacity

Water-holding capacity refers to the ability of a protein to absorb water and retain it against gravity within a protein matrix [93]. This retention depends on the conformation of the proteins, which disappears during hydrolysis. The peptides generated are unable to retain the same amount of water as the original protein [13]. However, it is known that FPHs can possess amino acids with hydrophilic polar groups, capable of increasing the water-holding capacity, favoring, for example, the cooking yield [93]. Alahmad et al. [49] obtained the highest WHC (water-holding capacity) value in bighead carp (*Hypophthalmichthys nobilis*) muscle FPHs produced with the highest DH (20.15%), establishing significant differences between values obtained with different DH (13.36% and 17.09%). This could be due to the increase in polar groups (NH_2_ and COOH) in FPHs. Dinakarkumar et al. [93] confirmed this with Pugnose Ponyfish (*Secutor insidiator*) flesh FPH. The WHC significantly decreased (*p* < 0.05) with the increasing FPH concentration (with papain and proteinase K and DH of 0.9 and 0.8, respectively) from 0.2% to 0.6%, while, from 0.6% to 1.0%, it slightly decreased. This was because the 1.0% FPHs had less exposed surface area, unlike the 0.2% FPHs, where the PHs presented the highest ratio between surface area and mass, obtaining the highest water retention capacity.

The OHC (oil-holding capacity) is a functional property that affects both the emulsifying and sensory properties [32], as it influences the taste of the final product. It is related to the hydrophobicity of the surface [48]. It has been shown that enzyme–substrate specificity and the DH influence the oil absorption capacity [56]. As the MW decreases, the OHC decreases because less oil is absorbed [49]. FPHs from by-products of Spanish mackerel (*Scomberomorus brasiliensis*) presented higher values with a lower DH [37]. However, Yathisha et al. [78] indicated that the oil-holding capacity increased as the hydrolysis time increased. In another study, Alahmad et al. [32] showed that the lowest OHC value (2.19 g/g FPH) was obtained with the highest DH (25.48%). This is because, as hydrolysis increases, proteins break down into smaller peptides. These lower MW peptides have a reduced capacity to retain oil, as their more compact and smaller structure is not as efficient at trapping oil compared to larger proteins. Parvathy et al. [48] obtained results of 2.24 ± 0.12 g/g of FPH from red meat of tuna (*Euthynnus affinis*), indicating that the ability of proteins to absorb fat influences the taste of food products. This research also claims that the OHC increased during hydrolyzation within a certain time range, whereas it dropped on further hydrolyzation, probably due to the lower MW of the peptides generated. Kumari et al. [47] reported similar OHC values (2.2 g/g of FPH) in Pink Perch (*Nemipterus japonicus*) head and viscera PHs, suggesting that the low OHC of PHs increases its application in meat and confectionary industries.

### 3.6. FPH Applications in the Food Industry

Currently, consumers demand healthy diets rich in nutrients [97]. Given the various characteristics and properties of FPHs, several authors have evaluated the inclusion of these foods in food matrices.

Unnikrishnan et al. [97] fortified mayonnaise by partially replacing egg yolk. They produced tuna (*Thunnus albacares*) red muscle PH with a molecular weight > 10 kDa to improve the mayonnaise’s functionality. The authors subjected the fortified samples and the control to sensory and nutritional analysis, as well as evaluating the emulsion stability. They noted a brownish color and a fishy flavor, along with a significant increase in protein and a decrease in fat in the samples. Overall, the fortified mayonnaise had a higher emulsion capacity than the control sample. In another study, Lima et al. [98] fortified yogurt by adding muscle FPH from *Cynoscion guatucupa*. In this case, the authors used the enzyme Protamex^®^ in hydrolysis and performed microencapsulation through spray-drying. The results highlighted the addition of microencapsulated FPH to yogurt, successfully masking the characteristic fish flavor. Additionally, the final products exhibited antioxidant activity and angiotensin-converting enzyme (ACE) inhibition activity. However, the addition of the hydrolysate to the yogurts, both free and microencapsulated, resulted in a slight decrease in firmness and an increase in cohesiveness.

Khodaei et al. [99] enriched semolina pasta with FPH from blue whiting (*Micromesistius poutassou*) and evaluated its effects on the sensory and physicochemical properties of the pasta. They indicated that cooking time, swelling index, water absorption, and cohesion were reduced. However, protein content, adhesiveness, and firmness of the pasta increased. The authors considered FPHs as a potential ingredient in the production of protein-enriched pasta.

In order to reduce NaCl in foods, Cho et al. [100] fortified white bread with anchovy PHs. The FPH used contained peptides of low molecular weight (<1.3 kDa) and free amino acids. The authors highlighted high amounts of alanine (sweet taste) and glutamic acid (umami and salty taste), as well as good fermentation rates and specific volume of the resulting bread. However, increasing the concentration of FPH resulted in higher dough hardness and lower adhesiveness. Therefore, FPH can replace NaCl in white bread production, but further studies are needed to improve its quality.

Idowu et al. [14] fortified whole wheat salted crackers by incorporating salmon PHs. They conducted sensory, textural, and nutritional analysis and found that the addition of FPH did not affect water activity. The authors noted that samples with FPH were darker. Additionally, this foodstuff with a higher FPH concentration was more bitter. Fortification also influenced the composition, as proteins, fat, ash, and cholesterol increased while sugar and fiber decreased, and calcium and phosphorus increased. Overall, this research was considered relevant in increasing the nutritional value of salted crackers. In another study, fortified biscuits with “desalted” salmon PHs were developed [101,102]. The researchers made a set of samples (0, 5, 10, 15, 20, and 25 g FPH/100 g dough) and evaluated their nutritional, sensory, textural, and physicochemical properties. Their findings noted that the addition of FPH in the process improved the texture of the biscuits. Thus, incorporating “desalted” salmon FPH can supplement the deficiency of some essential amino acids in wheat without altering the sensory properties of the final product.

FPHs from sea bass (*Dicentrarchus labrax*) and bonito (*Thunnus alalunga*) heads have also been used in the fortification of biscuits. The incorporation of FPH into the biscuits produced nutritional enrichment, especially in protein, but also caused color changes, which were more intense due to the increase of the Maillard reaction, and changes in sensory perception, where higher intensities of color and toasted flavor were perceived but also fish flavors. Scanning electron microscopy made it possible to visualize differences in the internal structure, which could be related to differences found in the instrumental texture measurements (decreased hardness and increased fracturability) [40,103].

**Figure 3:**
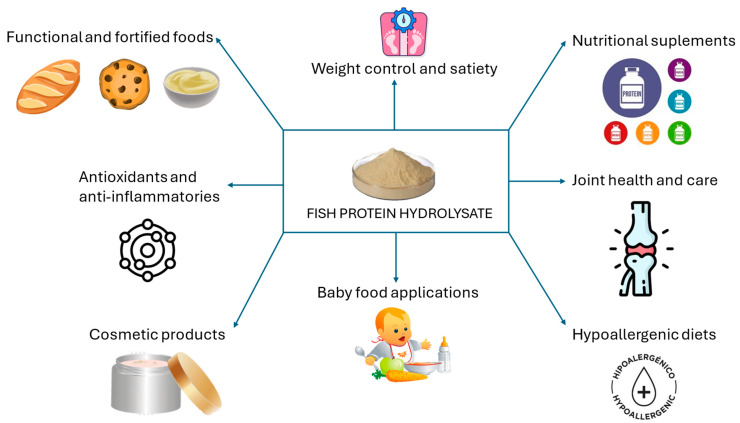
Current uses and future trends of fish protein hydrolysates.

The FPHs market is expected to reach USD 372.88 million by 2031, growing at a compound annual growth rate of 4.4% from USD 267.87 million in 2023. The rising demand for pharmaceutical products due to their rapid absorption, search for strategies to increase muscle mass, and ability to prevent certain diseases will drive market expansion, and, although fish protein hydrolysate is currently used in sectors such as animal feed, pet food, and cosmetics, increasing utilization in protein supplements, sports nutrition, and infant formulas is anticipated, which will propel the global market (Figure 3) [102]. In this sense, the first products based on fish hydrolysates are starting to be sold. Examples include dietary supplements in capsules (MOLVAL^®^) from common ling (*Molva molva*), capsules with antihypertensive effect (PeptACE^®^) from sarda (*Sarda* spp.), or sardine (*Sardina pilchardus*) PHs for food fortification (Valtyron^®^), which concludes that the product can be safe as a food ingredient at the proposed conditions of use and the proposed intake levels [104].

## 4. Conclusions

The conclusions drawn from the compilation of scientific data suggest that producing FPHs with acceptable color and without the characteristic fish flavors or odors require optimizing enzymatic hydrolysis conditions or employing masking techniques (deodorization, ultrafiltration techniques, use of adsorbents, etc.). This is undoubtedly an area where much research remains to be done. FPHs are known for their high nutritional value, with a high protein content and low lipid levels. While their potential health benefits for humans, such as antioxidant and antihypertensive effects, have been suggested, food safety remains a concern due to the lack of conclusive studies from official institutions, particularly when fish by-products are used. Nevertheless, the growing body of research on FPHs reflects increased interest in their application in food products and a trend toward better management of fish by-products to reduce environmental impact and increase their value. Additionally, more research is needed on the functional properties of FPHs and the optimal hydrolysis conditions to preserve these benefits. This could pave the way for the development of food enrichment, fortification products, and supplements that have a positive impact on consumer health in the near future.

## Figures and Tables

**Table 1:** CIEL*a*b* values obtained as a function of degree of hydrolysis (DH), enzyme, and fish species. Different letters in the same column denote statistically significant differences (*p* < 0.05) between degrees of hydrolysis for a given CIEL*a*b* coordinate.

DH (Time)	Color			Enzyme	Species	Authors
	L*	a*	b*			
**16.56% (1 h)**	86.89 ± 0.52 ^a^	1.39 ± 0.08 ^b^	15.81 ± 0.37 ^b^	Flavourzyme^®^	Bighead Carp (*Hypophthalmichthys nobilis*)	[32]
**22.23% (3 h)**	84.06 ± 0.24 ^b^	1.43 ± 0.05 ^b^	17.42 ± 0.18 ^a^			
**25.48% (6 h)**	83.98 ± 0.16 ^b^	1.92 ± 0.11 ^a^	17.97 ± 0.21 ^a^			
**5.3% (0.25 h)**	84.33 ± 0.02 ^a^	−0.54 ± 0.02 ^a^	22.13 ± 0.04 ^a^	Savinase^®^	Hake (*Merluccius merluccius*)	[31]
**6.5% (0.5 h)**	84.16 ± 0.03 ^a^	−0.48 ± 0.01 ^a^	21.1 ± 0.02 ^b^			
**7.7% (1 h)**	82.71 ± 1.10 ^a^	−0.50 ± 0.04 ^a^	15.16 ± 0.25 ^c^			
**8.6% (2 h)**	79.34 ± 2.80 ^b^	−0.31 ± 0.04 ^b^	13.47 ± 0.28 ^d^			
**31.59% (4 h)**	90.62 ± 0.05	−0.61 ± 0.01	17.16 ± 0.11	Papain	Tuna (*Thunnus albacares*)	[35]
**13.36% (1 h)**	89.23 ± 0.08 ^a^	0.17 ± 0.01 ^b^	10.96 ± 0.56 ^b^	Ficin	Bighead Carp (*Hypophthalmichthys nobilis*)	[49]
**17.09% (3 h)**	88.82 ± 0.10 ^b^	0.23 ± 0.05 ^ab^	12.29 ± 0.10 ^a^			
**20.15% (6 h)**	86.55 ± 0.09 ^c^	0.31 ± 0.06 ^a^	12.68 ± 0.03 ^a^			

**Table 2:** Comparison of the proximate composition among fish protein hydrolysates (FPHs) and raw material of different fish species. RM: raw material; FPH: fish protein hydrolysate.

Authors	Species	RM/FPH	Moisture	Protein	Fat	Ash
[51]	Caspian kutum (*Rutilus kutum*) by-products mixture	RM	78.88	15.1	4.73	2.19
		FPH	7.52	87.38	1.61	3.95
[48]	mackerel tuna (*Euthynnus affinis*) muscle	RM	-	28.25	-	-
		FPH	1.35	89.90	0.06	4.03
[52]	Liza (*Liza klunzingeri*) muscle	RM	73.36	22.46	2.21	9.52
		FPH	1.87	87.84	0.77	2.00

**Table 3:** Amino acid composition of different fish protein hydrolysates produced from distinct species and applying different enzymes. ALC: Alcalase^®^; FLA: Flavourzyme^®^; GI: gastrointestinal digestion.

Authors	[53]	[54]	[54]	[55]	[34]	[34]	[56]
Species	Nile Tilapia (*Oreochromis niloticus*) Viscera	Indian Mackerel (*Rastrelliger kanagurta*) Skin and Head	Indian Mackerel (*Rastrelliger kanagurta*) Skin and Head	Sardine (*Sardina pilchardus*) by-Products Mixture	Salmon (*Salmo salar*) Frames	Salmon (*Salmo salar*) Frames	Monkfish (*Lophius vomerinus*) Heads
Origin	Freshwater	Seawater	Seawater	Seawater	Seawater	Seawater	Seawater
Enzyme	ALC	ALC	FLA	GI	ALC	FLA	ALC
Histidine	2.04	3.55	3.29	5.20	3.40	3.58	1.40
Isoleucine	1.56	4.05	5.05	1.20	3.76	3.33	5.39
Leucine	2.19	7.35	6.99	4.85	7.06	6.39	8.47
Lysine	2.82	7.99	7.19	3.02	8.23	8.15	12.2
Methionine	0.88	3.15	3.05	1.52	3.14	2.83	3.42
Phenylalanine	1.07	4.25	4.15	3.02	3.55	2.99	4.15
Tyrosine	1.42	3.55	3.28	2.52	3.11	2.27	3.49
Threonine	1.26	3.95	4.15	-	4.56	4.13	4.49
Tryptophan	0.42	-	-	0.36	0.69	0.48	-
Arginine	1.93	7.55	8.21	2.90	6.52	6.56	5.22
Valine	2.78	5.25	5.45	2.58	4.48	4.17	5.50
Asparagine + aspartate	3.15	7.99	6.98	-	9.59	9.22	10.1
Glutamine + glutamate	3.85	12.55	11.79	11.52	14.13	14.65	14.0
Serine	1.19	4.59	4.69	-	4.61	4.62	4.98
Glycine	1.27	5.99	5.25	4.90	9.03	10.93	6.45
Alanine	1.56	4.98	4.25	9.52	6.75	7.19	5.92
Proline	0.99	5.29	4.99	3.10	5.14	5.53	4.37
Cysteine	0.32	0.95	0.99	2.44	0.01	0.00	-

**Table 4:** Antimicrobial activity shown by different fish protein hydrolysates produced from distinct species and using several enzymes. The results are shown as the percentage inhibition or as mm of the inhibition halo produced. NI: no inhibition; ALC: Alcalase^®^; PRO: Protamex^®^; FLA: Flavourzyme^®^; PEP: Pepsin.

Authors	Species	Enzymes	Fractionation	*L. monocytogenes*	*L. innocua*	*S. aureus*	*B. cereus*	*E. coli*	*S. enterica*	*S. typhimurium*	*Pseudomonas*
[80]	skipjack tuna (*Katsuwonus pelamis*)	ALC	head	3 mm		2.17 mm	1.17 mm	1.67 mm	1.83 mm	1.83 mm	
		ALC	bones	2 mm		2.17 mm	2.00 mm	2.17 mm	3.17 mm	2.67 mm	
		ALC	skin	2.67 mm		3.00 mm	1.33 mm	2.33 mm	3.17 mm	2.00 mm	
[23]	Yellowfin tuna (*Thunnus albacores*) viscera	PRO	<30 kDa	95%		82%		80%			78%
		PRO	10–30 kDa	60%		46%		50%			50%
		PRO	3–10 kDa	92%		72%		85%			83%
		PRO	<4 kDa	100%		97%		95%			95%
[54]	Indian mackerel (*Rastrelliger kanagurta*)	ALC (10 min)	Skin/head			13–18 mm		<7 mm			
		ALC (20 min)				>18 mm		7–13 mm			
		ALC (30 min)				>18 mm		13–18 mm			
		FLA (10 min)				<7 mm		<7 mm			
		FLA (20 min)				13–18 mm		<7 mm			
		FLA (30 min)				>18 mm		7–13 mm			
[77]	Whole common Kilka fish (*Clupeonella cultriventris caspi*)	PEP (30 min)	10–30 Da		NI	NI		NI	NI		
		PEP (60 min)			NI	32%		23%	34%		
		PEP (90 min)			NI	77%		NI	NI		
		PRO (30 min)			NI	NI		NI	NI		
		PRO (60 min)			NI	NI		NI	NI		
		PRO (90 min)			NI	56%		18%	NI		

**Table 5:** Literature in which the solubility of fish protein hydrolysates produced using different fish species, degrees of hydrolysis, and pH has been evaluated. ALC: Alcalase^®^; PRK: Proteinase K^®^; PAP: papain; FLA: Flavourzyme^®^.

Authors	Species	Enzymes	Degree of Hydrolysis (%)	pH	Main Results
[94]	Skipjack Tuna (*Katsuwonus pelmamis*) viscera	ALC	20%	3, 5, 7 and 9	Solubility increased directly proportional to pH (91.89%, 96.39%, 97.65%, and 100% respectively).
[47]	*Pink* *Perch* (*Nemipterus japonicus*) head and viscera		15.5%	2, 4, 6, 8 and 10	Solubility increased from 89.8 to 99.7 when hydrolysis pH increased.
[78]	Ribbon Fish (*Lepturacanthus savala*) viscera		19–41	2–12	Solubility increases as pH does, but at the isoelectric point (pH 4.4–5.5), solubility decreases deeply. Solubility increased 50% when comparing the one with the least degree of hydrolysis and the highest one.
[70]	Bighead Carp (*Hypophthalmichthys nobilis*) viscera		-	3, 5, 7 and 9	Solubility decreased at the isoelectric point. Fractionation allowed to see how small peptides (<3 kDa) showed higher solubility.
[93]	Secutor insidiator (*Pugnose ponyfish*) flesh	PRK and PAP	8 and 9	2, 4, 6, 7, 8 and 10	Solubility increased 69% when comparing native protein and FPH from both enzymes.
[32]	Bighead Carp (*Hypophthalmichthys nobilis*) whole fish	FLA	16.56–22.23 and 25.48	6	The highest solubility value (97.4%) was found at the highest degree of hydrolysis (25.48%).
[34]	Salmon (*Salmo salar*) frames	ALC and FLA	26.88 and 25.02	7	Debittering with alcohols resulted in a reduction of solubility. The decrease in alkalase FPH was 3 and 5.7% using 2-butanol and iso-propanol, respectively.

## Data Availability

The raw data supporting the conclusions of this article will be made available by the authors on request.
